# Domain-specific and generalised motivational pathways to STEM degree attainment: a 10-year longitudinal mediation analysis

**DOI:** 10.3389/fpsyg.2026.1898011

**Published:** 2026-08-17

**Authors:** Meicheng Yang

**Affiliations:** School of Humanities and Social Sciences, Jiangsu University of Science and Technology, Zhenjiang, China

**Keywords:** domain specificity, educational expectations, expectancy-value theory, longitudinal, math self-efficacy, secondary education, STEM degree, utility value

## Abstract

Understanding how adolescent motivational beliefs relate to long-term STEM degree attainment is a central question in educational psychology. Drawing on expectancy-value theory, this study examined whether math self-efficacy (a domain-specific belief) and academic utility value (a generalised belief about the instrumental importance of education) in Grade 10 were associated with STEM degree attainment at age 26, and whether educational expectations in Grade 12 mediated these associations. Data came from the Education Longitudinal Study of 2002, a nationally representative U.S. sample followed from 2002 to 2012 (*N* = 16,197). Structural equation modelling yielded two main findings. First, math self-efficacy was associated with STEM attainment primarily through a direct pathway, with its indirect pathway via expectations being smaller in magnitude. Second, academic utility value exhibited full mediation: its direct association with STEM attainment was not distinguishable from zero, whereas its indirect association through educational expectations was significant and accounted for approximately 85% of the total association. Family socioeconomic status was associated with educational expectations but showed no direct association with STEM attainment. These findings were robust across ten supplementary analyses, including one that included Grade 10 educational expectations as an additional covariate. The available items confound domain anchoring with the expectancy–value distinction, so findings are discussed with respect to both interpretations. These results suggest that efforts to broaden STEM participation may benefit from addressing domain-specific competence beliefs alongside students’ broader educational ambitions.

## Introduction

The underrepresentation of qualified graduates in science, technology, engineering, and mathematics (STEM) fields remains a persistent challenge for post-industrial economies. Understanding why some students complete STEM degrees while others do not—despite comparable baseline ability—requires examining not only structural factors but also the psychological mechanisms that channel adolescent motivation into long-term educational outcomes.

Eccles’ expectancy-value theory (EVT) provides a framework for understanding how motivational beliefs relate to educational choices and achievement (Eccles and Wigfield, 2002). The theory holds that two classes of psychological factors jointly predict educational outcomes: expectancy beliefs, which capture confidence in one’s ability to succeed in a given domain, and subjective task values, which capture the perceived importance, interest, and utility of that domain (Eccles et al., 1983). Contemporary formulations of the theory further specify that these beliefs are shaped by developmental contexts and influence behaviour through a hierarchy of proximal and distal mediators (Eccles and Wigfield, 2020).

Within this framework, educational expectations—the level of education an individual realistically plans to attain—occupy a conceptually important position. For clarity, we use the term expectancy beliefs to refer to the confidence component of EVT (e.g., self-efficacy) and the term educational expectations to refer to the level of education one plans to attain. These are distinct constructs within EVT: the former is a motivational input, the latter a cognitive mediator that may channel the influence of motivational beliefs toward educational outcomes. EVT posits that expectancy and value beliefs do not translate directly into educational choices. Rather, they first shape the cognitive representation of one’s educational future (Eccles, 1994), which in turn guides domain-specific decisions such as whether to pursue a STEM degree. This mediating role is theoretically plausible because STEM degrees are, by definition, high-educational-attainment outcomes: entering a STEM profession typically requires at least a bachelor’s degree, and many STEM careers require graduate education. Educational expectations may therefore function as a necessary, though not sufficient, condition for STEM pursuit—a student who has not formed the expectation of completing college is unlikely to enter the STEM pipeline regardless of their interest in science or mathematics.

We propose a division of theoretical labor between the two pathways in our model. Domain-specific math self-efficacy, by virtue of its explicit anchoring in mathematics, provides the directional signal that biases students toward quantitative fields rather than toward other high-educational-attainment domains such as law or medicine. Educational expectations, by contrast, provide the educational-level gateway through which this directional signal must pass. This gateway function follows from the institutional structure of STEM careers rather than from motivational psychology per se: because STEM professions require postsecondary credentials, the formation of college expectations is a necessary precondition for entering the STEM pipeline, regardless of the strength of one’s mathematical interest or confidence. EVT directly supports the first link in our model—motivational beliefs shape educational expectations (Eccles, 1994; Eccles and Wigfield, 2002)—but the second link—educational expectations predict STEM attainment—derives its theoretical rationale from the structural characteristics of STEM occupations rather than from the expectancy-value framework alone. The present study tests whether educational expectations function as a transmission mechanism that channels the effects of both domain-specific and generalised motivational beliefs toward a structurally gated outcome.

Empirical research supports the broader role of educational expectations as a mediator between social origins and educational outcomes. Studies across multiple national contexts have shown that educational expectations mediate the association between family socioeconomic status and later academic achievement and attainment (Yong et al., 2026; Xu and Fu, 2024; Hofer et al., 2024). Moreover, educational expectations have been empirically linked to STEM-specific outcomes: prior longitudinal research has documented that higher educational expectations predict STEM degree attainment (Maltese and Tai, 2011) and that the developmental trajectories of educational expectations and science performance jointly predict STEM degree completion (Yeung and Igarashi, 2025). However, this mediation framework has rarely been extended to examine whether educational expectations similarly channel the effects of motivational beliefs—as distinct from family background—on domain-specific outcomes such as STEM degree attainment.

The present study focuses on two motivational beliefs that EVT identifies as central: math self-efficacy, a domain-specific expectancy belief, and academic utility value, a generalised belief about the instrumental importance of education. These two constructs differ in their domain anchoring in ways that may carry implications for how they relate to STEM outcomes. Math self-efficacy refers specifically to confidence in performing mathematical tasks (Bandura, 1997) and has been shown to predict STEM-related choices as early as middle school (Cuder et al., 2024). Utility value, as measured in the available data, refers to education in general rather than to mathematics specifically—a measurement characteristic that is revisited in the Discussion as an interpretive boundary condition. Prior research has documented that both types of motivational beliefs are already present and predictive of academic choices before Grade 10 (Simpkins et al., 2006; Granello et al., 2025), yet they remain malleable during the high school years, when college preparation activities intensify and educational plans are revised (Ozturk et al., 2024). Educational expectations themselves exhibit meaningful instability during this period (Dräger and Burger, 2026; Morgan, 2005), indicating that the high school years represent a developmental window in which motivational beliefs may shape the formation and revision of educational plans.

Several characteristics of the existing literature leave important gaps. Most longitudinal studies of STEM motivation track participants for one to 4 years and capture proximal outcomes such as STEM intentions rather than actual degree attainment (Lee et al., 2024; Jiang et al., 2020). Many rely on convenience samples from single institutions or regional school districts (e.g., Robinson et al., 2022), and educational expectations are frequently included as covariates rather than as formally tested mediators (Morris, 2016; Adamuti-Trache and Zhang, 2023). Although EVT-grounded research has examined expectancy-value profiles (Gaspard et al., 2019; Fong et al., 2021; Fong and Kremer, 2020; Wille et al., 2020; Saw et al., 2025) and documented the developmental trajectories of utility value (Petersen and Hyde, 2017), few studies have combined expectancy and value constructs in a single formal mediational model spanning the full secondary-to-postsecondary transition. The few nationally representative, long-term studies in this area have not incorporated both expectancy and value constructs as joint predictors mediated by educational expectations (Bohrnstedt et al., 2023; Kurban and Cabrera, 2020; Zhao and Perez-Felkner, 2022; Yeung and Igarashi, 2025; Guo et al., 2015).

The present study was designed to address these gaps using the Education Longitudinal Study of 2002 (ELS:2002; Ingels et al., 2004, 2014), a nationally representative U.S. sample followed from Grade 10 (2002) to approximately age 26 (2012). The U.S. educational system is characterised by considerable flexibility in high-school course selection, multiple pathways into postsecondary education, and a relatively open admission structure at many institutions—features that make educational expectations particularly relevant as a mediator, because the link between motivation and attainment depends heavily on students’ own planning and choice rather than on formal tracking decisions. We examined a structural equation model linking Grade 10 math self-efficacy and academic utility value to STEM degree attainment at age 26, with Grade 12 educational expectations as a mediator, controlling for baseline math ability, family socioeconomic status, gender, race/ethnicity, and—in a supplementary model—Grade 10 educational expectations. We hypothesised that (H1) math self-efficacy would positively predict educational expectations, (H2) utility value would positively predict educational expectations, (H3) educational expectations would positively predict STEM degree attainment, (H4) math self-efficacy would have a significant indirect association with STEM degree attainment through educational expectations, and (H5) utility value would have a significant indirect association with STEM degree attainment through educational expectations.

## Materials and methods

### Data and sample

ELS:2002 employed a two-stage stratified probability sampling design (Ingels et al., 2004, 2014). The base-year sample comprised 15,362 Grade 10 students assessed in spring 2002. The sample was refreshed in 2004 to maintain national representativeness, yielding a total analytic sample of 16,197. Follow-up surveys were administered in 2004 (Grade 12), 2006, and 2012. The present study used data from 2002, 2004, and 2012.

Different analyses used different subsamples, depending on the estimation method and treatment of missing data. The confirmatory factor analysis (CFA) for math self-efficacy used listwise deletion, retaining the 10,443 students with complete data on all five self-efficacy items; the two-factor CFA used 10,253 students with complete data on all eight motivational items. The primary generalised structural equation model (GSEM) used full-information maximum likelihood (FIML), yielding an effective analytic *N* of 14,527. The balanced repeated replication (BRR)-weighted factor-score models excluded the 3,065 observations with panel weight zero, yielding *N* = 10,039. The factor-score logit models used listwise deletion across all covariates, resulting in *N* = 7,581. These sample-size differences reflect the properties of the respective estimators and do not represent differential inclusion criteria.

Missing data in the primary GSEM were handled via FIML under the assumption that data were missing at random conditional on the variables in the model (Enders, 2010; Graham, 2009). Because the model included the strongest known predictors of panel attrition—baseline math achievement, family socioeconomic status (SES), gender, and race/ethnicity—the missing at random (MAR) assumption was considered tenable (Schafer and Graham, 2002). BRR-weighted estimates incorporating a panel-weight adjustment for differential non-response were compared with FIML estimates as a sensitivity check (Solon et al., 2015).

### Measures

Table 1 provides a mapping between the original ELS:2002 variable codes used in the analyses and the substantive item content.

**Table 1:** Mapping between original ELS:2002 variable codes and item labels.

Original ELS code	Construct	Short label	Full item wording	Response scale
BYS89A	Math self-efficacy	MSE1	“I’m confident that I can do an excellent job on my math tests”	1–4 Likert
BYS89B	Math self-efficacy	MSE2	“I’m certain I can understand the most difficult material presented in math texts”	1–4 Likert
BYS89L	Math self-efficacy	MSE3	“I’m confident I can understand the most complex material presented by my math teacher”	1–4 Likert
BYS89R	Math self-efficacy	MSE4	“I’m confident I can do an excellent job on my math assignments”	1–4 Likert
BYS89U	Math self-efficacy	MSE5	“I’m certain I can master the skills being taught in my math class”	1–4 Likert
BYS89D	Academic utility value	UV1	“I study to get a good job”	1–4 Likert
BYS89H	Academic utility value	UV2	“I study to increase my job opportunities”	1–4 Likert
BYS89P	Academic utility value	UV3	“I study to ensure that my future will be financially secure”	1–4 Likert
F1STEXP	Educational expectations	EdExp	“As things stand now, how far in school do you think you will get?”	1–8 ordinal
F3TZSTEM1CRED	STEM degree attainment	STEM degree	Transcript-verified STEM credential (SMART Grant classification)	0/1 binary
BYTXMSTD	Baseline math achievement	Math achievement	Standardised mathematics test score	*T*-score
BYSES1	Family SES	Family SES	NCES composite of parental education, occupation, and income	*z*-score
BYSEX	Gender	Gender	Student’s sex (school records)	1 = male, 2 = female
BYRACE	Race/ethnicity	Race/ethnicity	Student’s self-identified race/ethnicity (composite)	7 categories

#### Math self-efficacy

In Grade 10, students rated five items on a 4-point Likert scale (1 = *almost never*, 4 = *almost always*). The items assessed confidence in performing mathematics tasks (e.g., “I’m confident that I can do an excellent job on my math tests”; “I’m certain I can understand the most difficult material presented in math texts”; “I’m confident I can understand the most complex material presented by my math teacher”; “I’m confident I can do an excellent job on my math assignments”; “I’m certain I can master the skills being taught in my math class”). CFA supported a single-factor structure (standardised loadings 0.81–0.90; *α* = 0.932). The five items were used as indicators of a latent math self-efficacy factor. Use of ELS:2002 items to measure math-related self-beliefs is well established (Nix et al., 2015; Peguero and Shaffer, 2015; Saltiel, 2023; Sublett and Gottfried, 2017).

#### Academic utility value

Three items assessed the instrumental value students attached to education (e.g., “I study to get a good job”; *α* = 0.847; loadings 0.76–0.87). Items used the same 4-point Likert scale as the self-efficacy measure. Because these items refer to education in general rather than to mathematics specifically, the utility value measure is appropriately interpreted as a generalised motivational belief—an interpretive boundary condition that is revisited in the Discussion.

#### Educational expectations

In Grade 12 (2004), students reported how far in school they expected to go on an 8-point ordinal scale (1 = *less than high school*, 8 = *PhD/MD*). Students responding “do not know” (*n* = 1,318) were treated as missing, with values estimated via FIML. This treatment was adopted because “do not know” responses are ambiguous regarding their position on the underlying continuum of educational expectations; supplementary analyses (Supplementary Table S5) confirmed that the pattern of results was unchanged when these responses were handled via complete-case analysis or recoded to the lowest category. The use of ELS:2002 educational expectations as a key construct in longitudinal research is well documented (Adamuti-Trache and Zhang, 2023; Morris, 2016). In a supplementary sensitivity analysis, Grade 10 educational expectations were included as an additional covariate to strengthen the temporal ordering of the mediation chain. Grade 10 and Grade 12 educational expectations showed a modest positive correlation (*r* = 0.19, *p* < 0.001, *N* = 12,036), consistent with the known variability of educational expectations during the high-school years, when college preparation activities intensify and plans are revised (Dräger and Burger, 2026; Morgan, 2005). This modest stability indicates that the majority of the variance in Grade 12 expectations is not attributable to baseline expectations and is thus available to be accounted for by motivational beliefs and other covariates.

#### STEM degree attainment

At the 2012 follow-up, postsecondary transcript data identified whether students had earned a STEM credential by June 2013. STEM fields were defined according to the National Science and Mathematics Access to Retain Talent (SMART) Grant Program (2006) (SMART Grant) classification (34 C.F.R. § 691.17(d)), using the 2010 Classification of Instructional Programs (CIP) codes. The specific CIP codes classified as STEM under this regulation are documented in the ELS:2002 Third Follow-Up Data File Documentation (Ingels et al., 2014) and are available from the U.S. Government Publishing Office. The outcome was coded dichotomously (1 = STEM degree, *n* = 1,158; 0 = otherwise, *n* = 10,253; Zhang et al., 2019; Saltiel, 2023).

#### Covariates

Baseline math achievement (*T*-score, *M* = 50.71, SD = 9.91), family SES (NCES composite, *M* = −0.12, SD = 1.08), gender, and race/ethnicity (White, Black, Hispanic, Asian, Other) were included. Achievement and SES were standardised to *z*-scores.

### Analytic strategy

#### Measurement model

CFAs were evaluated against conventional thresholds (comparative fit index (CFI) and Tucker-Lewis index (TLI) ≥ 0.95, standardized root mean square residual (SRMR) ≤ 0.08; Hu and Bentler, 1999). Given the large sample and small degrees of freedom, greater emphasis was placed on CFI and SRMR than on root mean square error of approximation (RMSEA) (Kenny et al., 2015). Modification indices (MI) were inspected for theoretically defensible residual correlations. A two-factor CFA including both motivational constructs was conducted to evaluate discriminant validity; a likelihood-ratio test compared this model with a one-factor model constraining all eight items to load on a single factor.

#### Structural equation model

A GSEM was specified with two equations: an ordered logit link for educational expectations, and a logit link for STEM degree attainment. Both latent motivational factors, all covariates, and residual covariances reflecting shared item wording (between the two evaluation-type math self-efficacy items, MSE1 and MSE2, and between the two completion-type items, MSE4 and MSE5) were included in the model. All path coefficients are unstandardised logit coefficients; odds ratios (ORs) are reported where appropriate. FIML was used for missing data. The full GSEM estimated approximately 50 parameters; with 1,158 STEM degree earners, the events-per-variable ratio was approximately 23, well above the recommended minimum of 10 (Peduzzi et al., 1996; Vittinghoff and McCulloch, 2007).

#### Nested model comparisons

Three nested models were compared: Model 1 (core paths only), Model 2 (adding achievement and SES), and Model 3 (adding gender and race/ethnicity). Particular attention was paid to the math self-efficacy (MathSE) → educational expectations path. To determine which covariate drove the coefficient recovery from M2 to M3, supplementary regressions separately added gender and race/ethnicity to M2.

#### Indirect and total associations

**Figure 1:**
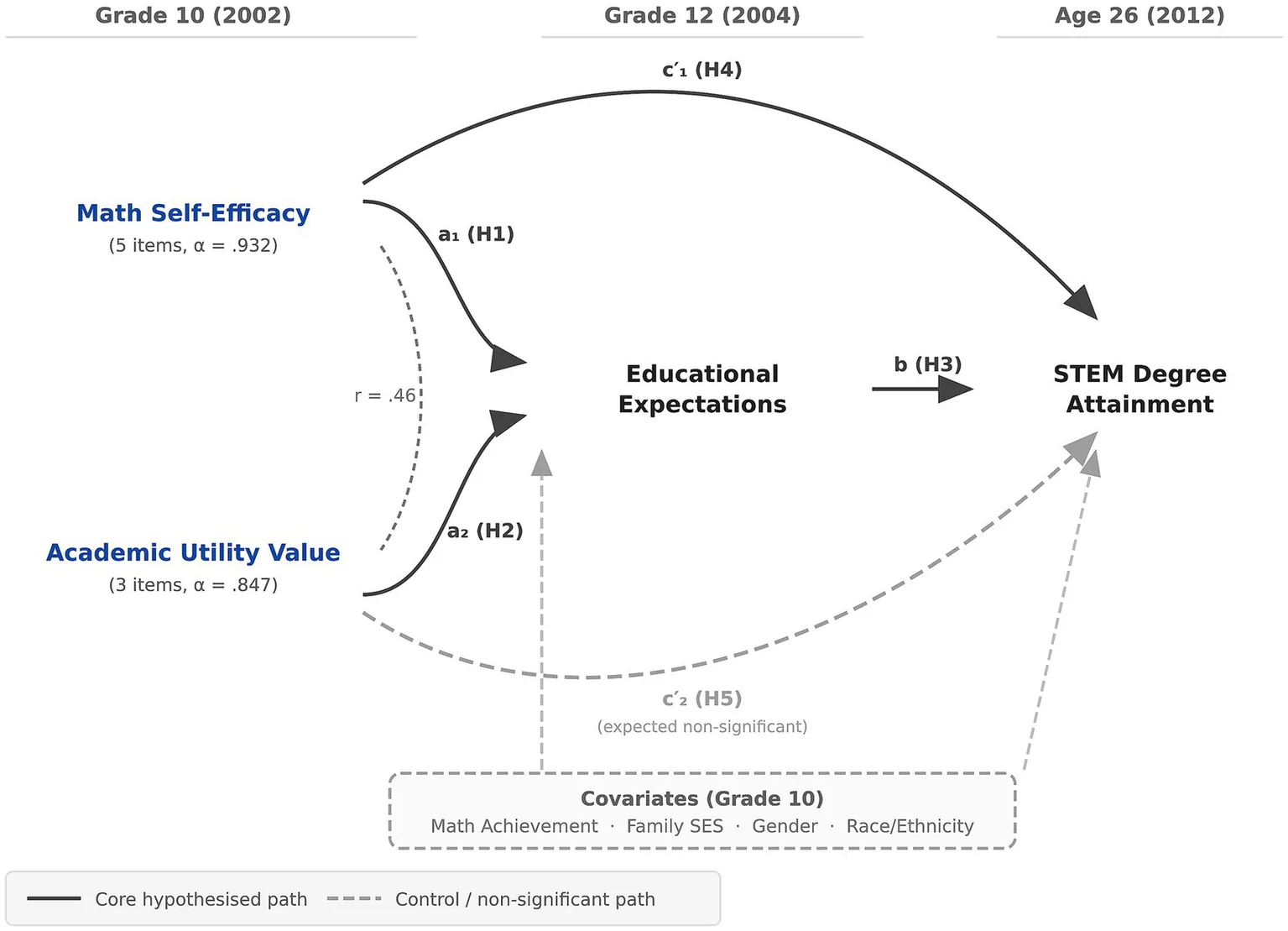
Conceptual model of the hypothesised structural paths. Solid lines indicate core hypothesised paths; dashed lines indicate control paths or expected non-significant paths. MathSE, math self-efficacy; UtilVal, academic utility value; EdExp, educational expectations.

Indirect associations were computed as the product of the a and b paths, with standard errors obtained via the delta method. Total associations were computed as the sum of direct and indirect components. To separate genuine mediation from rescaling artefacts inherent to logit models—where the residual variance of the latent dependent variable is fixed at π^2^/3, causing coefficient estimates to change across nested specifications even when the underlying effects are unchanged—the Karlson-Holm-Breen (KHB) method was applied (Karlson et al., 2012). The KHB decomposition was conducted on factor-score logit models with educational expectations as the mediator and all covariates as concomitants. Figure 1 presents a conceptual diagram of the hypothesised paths.

#### Robustness and sensitivity analyses

The robustness of the findings was evaluated through a series of supplementary analyses, including weighted estimation, alternative model specifications, sensitivity to missing-data treatments, and tests for unmeasured confounding. Full details of all procedures and results are provided in the Supplementary Tables S1–S11.

## Results

### Descriptive statistics and missing data

Approximately 35% of students had missing data on motivational items, primarily because some schools did not administer the full questionnaire. Educational expectations were missing for 16.0% and STEM degree data for 29.5%. Students with missing STEM data scored significantly lower on baseline math achievement (*d* = 0.80), family SES (*d* = 0.46), and motivational variables (ds = 0.23–0.32). Logistic regressions confirmed that low achievement, low SES, and male gender predicted data missingness (Supplementary Table S6); because these variables were included in all models, the MAR assumption was considered tenable. Approximately 10.1% of students with valid transcript data earned a STEM degree (*n* = 1,158; see Table 2).

**Table 2:** Descriptive statistics for analysis variables.

Variable	Valid N	M/%	SD	Min	Max
MSE1	11,391	2.55	0.93	1	4
MSE2	11,432	2.37	0.93	1	4
MSE3	11,047	2.47	0.96	1	4
MSE4	10,823	2.63	0.94	1	4
MSE5	10,661	2.67	0.94	1	4
UV1	11,304	2.73	0.93	1	4
UV2	11,119	2.67	0.97	1	4
UV3	10,928	2.73	0.96	1	4
EdExp	13,612	—	—	1	8
Less than HS	50	0.4%			
GED	183	1.3%			
HS only	674	5.0%			
2-year college	2,049	15.1%			
Some college	504	3.7%			
Bachelor’s	4,776	35.1%			
Master’s	3,283	24.1%			
PhD/MD	2,093	15.4%			
STEM degree (yes)	1,158	10.1%			
Math achievement	15,892	50.71	9.91	19.38	86.68
Family SES	15,892	−0.12	1.08	−4.00	1.82
Female	7,717	50.2%			

### Confirmatory factor analysis

The initial single-factor CFA for math self-efficacy showed mixed fit (CFI = 0.960, TLI = 0.920, RMSEA = 0.182, SRMR = 0.030). Modification indices revealed two large residual correlations: between items involving evaluation of math materials (MI = 1,095) and between items involving completion-oriented tasks (MI = 867). After adding these residual covariances, fit improved substantially [CFI = 0.993, TLI = 0.976, RMSEA = 0.099 (90% confidence interval (CI): 0.090, 0.108), SRMR = 0.011]. Standardised loadings ranged from 0.81 to 0.90 (all *p* < 0.001), with *α* = 0.932.

The three-item utility value CFA was exactly identified; standardised loadings ranged from 0.76 to 0.87. The two-factor CFA demonstrated good fit (CFI = 0.987, TLI = 0.979, RMSEA = 0.065 (90% CI: 0.061, 0.069), SRMR = 0.028) with a latent correlation of r = 0.46 (*p* < 0.001). A one-factor model fit significantly worse (Δ*χ*^2^(1) = 10,338, *p* < 0.001) (see Table 3).

**Table 3:** Confirmatory factor analysis results.

Parameter	Math SE (no residuals)	Math SE (with residuals)	Two-factor CFA
Standardised loadings			
MSE1	0.829	0.807	0.806
MSE2	0.836	0.826	0.821
MSE3	0.876	0.896	0.894
MSE4	0.876	0.855	0.863
MSE5	0.872	0.851	0.859
UV1			0.767
UV2			0.858
UV3			0.806
Residual covariances			
e.MSE1 ↔ e.MSE2	—	0.312	0.094
e.MSE4 ↔ e.MSE5	—	0.295	0.061
Latent correlation (MathSE–UtilVal)			0.464
Model fit			
*χ*^2^ (df)	1734.81 (5)	309.82 (3)	762.00 (17)
CFI	0.960	0.993	0.987
TLI	0.920	0.976	0.979
RMSEA [90% CI]	0.182 [0.175, 0.189]	0.099 [0.090, 0.108]	0.065 [0.061, 0.069]
SRMR	0.030	0.011	0.028
Cronbach’s α		0.932	0.847 (UtilVal)

All standardised loadings are significant at *p* < 0.001. For the two-factor CFA, *N* = 10,253 (listwise). The one-factor model fits significantly worse: Δ*χ*^2^(1) = 10,338, *p* < 0.001.

### Structural equation model

The full GSEM converged (*N* = 14,527). Path coefficients are presented in Table 4.

**Table 4:** Path coefficients from the generalised structural equation model.

Path	*b* (SE)	*z*	*p*	OR [95% CI]
EdExp (ordered logit)				
Math self-efficacy → EdExp	0.114 (0.032)	3.55	< 0.001	—
Utility value → EdExp	0.539 (0.034)	16.04	< 0.001	—
Math achievement (z) → EdExp	0.658 (0.021)	31.63	< 0.001	—
Family SES (z) → EdExp	0.701 (0.028)	25.42	< 0.001	—
Female →EdExp	0.671 (0.033)	20.07	< 0.001	—
STEM degree (logit)				
EdExp → STEM degree	0.389 (0.039)	10.00	< 0.001	1.48 [1.37, 1.59]
Math self-efficacy → STEM degree	0.370 (0.066)	5.61	< 0.001	1.45 [1.27, 1.65]
Utility value → STEM degree	0.037 (0.067)	0.55	0.581	1.04 [0.91, 1.18]
Math achievement (z) → STEM degree	0.704 (0.050)	14.07	< 0.001	2.02 [1.83, 2.23]
Family SES (z) → STEM degree	−0.004 (0.059)	−0.07	0.944	1.00 [0.89, 1.12]
Female → STEM degree	−0.671 (0.073)	−9.13	< 0.001	0.51 [0.44, 0.59]

*N* = 14,527 (GSEM analytic sample). Coefficients are unstandardised logit coefficients. OR, odds ratio; CI, confidence interval. The latent correlation between math self-efficacy and utility value is *r* = 0.46. Race/ethnicity coefficients appear in Supplementary Table S1.

#### Educational expectations

Math self-efficacy (*b* = 0.114, *p* < 0.001) and utility value (*b* = 0.539, *p* < 0.001) positively predicted educational expectations, supporting H1 and H2. Baseline math achievement (*b* = 0.658, *p* < 0.001) and family SES (*b* = 0.701, *p* < 0.001) were the strongest predictors. Female students reported higher expectations (*b* = 0.671, *p* < 0.001). Black (*b* = 0.341, *p* < 0.001), Hispanic (b = 0.196, *p* < 0.001), and Asian (*b* = 0.743, *p* < 0.001) students reported higher educational expectations than White students, net of SES and achievement. Full coefficients for all covariates appear in Supplementary Table S1.

#### STEM degree attainment

Educational expectations significantly predicted STEM degree attainment [*b* = 0.389, *p* < 0.001; OR = 1.48, 95% CI (1.37, 1.59)], supporting H3. Math self-efficacy showed a significant direct association [*b* = 0.370, *p* < 0.001; OR = 1.45, 95% CI (1.27, 1.65)]. Utility value showed no significant direct association [*b* = 0.037, *p* = 0.581; OR = 1.04, 95% CI (0.91, 1.18)]. Baseline math achievement was the strongest predictor (*b* = 0.704, *p* < 0.001), whereas family SES was not significant (*b* = −0.004, *p* = 0.944). Female students were substantially less likely to earn a STEM degree (*b* = −0.671, *p* < 0.001; Cheryan et al., 2017).

#### Sensitivity of the MathSE–expectations path

The path from math self-efficacy to educational expectations, on which its indirect association depends, showed sensitivity to covariate specification. In Model 1 (no covariates), this path was *b* = 0.360 (*p* < 0.001). In Model 2 (adding achievement and SES), it decreased to *b* = 0.025 (*p* = 0.428). In Model 3 (adding gender and race/ethnicity), it recovered to *b* = 0.114 (*p* < 0.001). Supplementary analyses identified gender as the primary driver of this recovery: when only gender was added to Model 2, the coefficient was *b* = 0.137 (*p* < 0.001); when only race/ethnicity was added, it was *b* = 0.052 (*p* = 0.015). This pattern is consistent with a suppression effect—male gender is associated with higher math self-efficacy but lower educational expectations, and controlling for gender removes variance in MathSE that is negatively correlated with expectations, revealing MathSE’s independent positive association. Full nested-model results appear in Supplementary Table S1. By contrast, the utility value → expectations path was robust across all specifications (M1: *b* = 0.580; M2: *b* = 0.621; M3: *b* = 0.539; all ps < 0.001).

#### Nested model—mediation

When educational expectations was removed from the model, the direct association of utility value with STEM degree attainment was significant (*b* = 0.148, *p* = 0.020), compared with b = 0.037 (*p* = 0.581) in the full model. Because logit coefficients are not directly comparable across nested specifications, this comparison was supplemented by KHB decomposition.

### Indirect and total associations

The indirect association of math self-efficacy was statistically significant but small (*b* = 0.044, *p* = 0.001, 10.7% of total), supporting H4 with qualification. The indirect association of utility value was larger and highly significant (*b* = 0.210, *p* < 0.001, 85.1% of total), supporting H5. Total associations were *b* = 0.414 (*p* < 0.001) for math self-efficacy and *b* = 0.247 (*p* < 0.001) for utility value (Table 5).

**Table 5:** Direct, indirect, and total associations with STEM degree attainment.

Association	*b* (SE)	*z*	*p*	% of total
Math self-efficacy				
Direct	0.370 (0.066)	5.61	< 0.001	89.3%
Indirect (via ed. expectations)	0.044 (0.013)	3.36	0.001	10.7%
Total	0.414 (0.067)	6.17	< 0.001	100%
Utility value				
Direct	0.037 (0.067)	0.55	0.581	14.9%
Indirect (via ed. expectations)	0.210 (0.025)	8.49	< 0.001	85.1%
Total	0.247 (0.067)	3.67	< 0.001	100%

Indirect associations computed via the delta method. KHB decomposition confirmed that the indirect association of utility value reflects genuine mediation rather than a rescaling artefact (rescaling factor = 1.15). The indirect association of math self-efficacy is small and the underlying MathSE → educational expectations path was not significant in Model 2 (controlling for achievement and SES). See text and Table S10 for details.

### KHB decomposition

For utility value, the reduced model (without mediator) yielded a significant total association (*b* = 0.118, *p* = 0.005), whereas the full model yielded a non-significant direct association (*b* = 0.037, *p* = 0.383). The rescaling factor was 1.15; after correcting for rescaling, the residual direct association remained non-significant, and the indirect association was significant (*b* = 0.081, *p* < 0.001). For math self-efficacy, rescaling was negligible (0.99), and the direct association remained significant (*b* = 0.298, *p* < 0.001). Full output appears in Supplementary Table S10.

### Gender differences

Gender-stratified GSEMs revealed that educational expectations were a significant predictor of STEM attainment for both females (*b* = 0.668, *p* < 0.001) and males (*b* = 0.248, *p* < 0.001). Supplementary analyses using average marginal effects (AME) and a linear probability model (LPM) were conducted because the baseline STEM participation rate differed substantially by gender. The AME of educational expectations on STEM degree attainment was 2.52 percentage points per one-unit increase for males (*p* < 0.001) and 4.40 percentage points for females (*p* < 0.001), a difference of 1.88 percentage points. In the LPM, the interaction between gender and educational expectations was not significant (*b* = −0.008, *p* = 0.125). In the logit specification, the interaction was significant (*b* = 0.428, *p* < 0.001; Wald test comparing independent subgroup coefficients: *z* = 4.98, *p* < 0.001). Taken together, these analyses indicate that the association between educational expectations and STEM degree attainment is descriptively stronger for females than for males, though the magnitude of this difference is modest and sensitive to the choice of scale.

### Robustness and sensitivity analyses

Full results of all robustness checks appear in Supplementary Tables S1–S11. The BRR-weighted model replicated all key findings: the direct association of utility value with STEM degree attainment remained non-significant in the weighted specification (*b* = 0.014, *p* = 0.785). In the college-attending subsample (*n* = 10,428), the direct association of utility value with STEM degree attainment was non-significant (*b* = −0.055, *p* = 0.435), ruling out the interpretation that the full-mediation pattern is an artefact of college-entry thresholds. Three alternative treatments of “do not know” responses produced substantively identical results. The *E*-value for educational expectations was 2.30, and for math self-efficacy was 2.03 (VanderWeele and Ding, 2017). Firth’s penalised logit produced coefficients nearly identical to standard logit estimates. The multinomial logit model confirmed that utility value predicted the college-entry contrast (*b* = −0.147, *p* < 0.001) but not the STEM vs. non-STEM contrast (*b* = −0.008, *p* = 0.858). When Grade 10 educational expectations were added as an additional covariate in the expectations equation of the GSEM, the pattern of associations was virtually unchanged: math self-efficacy (*b* = 0.119, *z* = 3.68, *p* < 0.001) and utility value (*b* = 0.529, *z* = 15.69, *p* < 0.001) remained significant predictors of Grade 12 expectations, and the direct association of utility value with STEM degree attainment remained non-significant (*b* = 0.030, *z* = 0.45, *p* = 0.652). Grade 10 expectations themselves significantly predicted Grade 12 expectations (*b* = 0.108, z = 7.32, *p* < 0.001). A supplementary factor-score logit model that additionally included Grade 10 expectations in the STEM degree equation found no significant direct association with STEM attainment (*b* = 0.036, *p* = 0.401), and all other paths remained unchanged. Taken together, these results indicate that the motivational beliefs measured in Grade 10 have an incremental association with educational expectations measured 2 years later, over and above the stability of expectations themselves.

## Discussion

This study examined the pathways through which adolescent motivational beliefs are associated with STEM degree attainment over a 10-year period, using a nationally representative U.S. longitudinal sample. Two main findings emerged.

### Divergent pathways for domain-specific and generalised motivational beliefs

The most robust finding concerns academic utility value: its association with STEM degree attainment was almost entirely indirect, via educational expectations. This pattern held across all three nested model specifications, both genders, the college-attending subsample, alternative missing-data treatments, and both unweighted and BRR-weighted estimation. The KHB decomposition confirmed that this pattern reflects genuine mediation rather than a rescaling artefact of the logit link.

Math self-efficacy showed a different pattern: a significant direct association with STEM degree attainment (*b* = 0.370, *p* < 0.001) alongside a small and model-sensitive indirect association (*b* = 0.044, *p* = 0.001). The path from math self-efficacy to educational expectations, on which the indirect association is based, was not significant when achievement and SES were controlled (*b* = 0.025, *p* = 0.428) and recovered only when gender was added to the model—a pattern consistent with suppression. The indirect pathway from math self-efficacy to STEM degree attainment was statistically significant but substantively small (10.7% of the total association) and was not robust to the inclusion of achievement and SES controls without gender in the model. These findings provide qualified support for H4.

#### Interpretive boundary condition

The math self-efficacy items are explicitly anchored to mathematics, whereas the utility value items refer to education in general. The divergent mediational patterns therefore confound two conceptually distinct dimensions: the expectancy–value distinction at the heart of EVT, and the domain-specific versus generalised distinction in measurement. It is possible that a domain-specific measure of utility value—for example, items such as “mathematics is useful for my future career”—would exhibit a pattern more similar to that of math self-efficacy. Conversely, a generalised measure of academic self-efficacy might behave more like the present utility value measure. The available data do not permit these two dimensions to be disentangled; the findings are appropriately interpreted as comparing domain-specific and generalised motivational beliefs rather than expectancy and value constructs per se.

With these measurement caveats in mind, the observed contrast between the two mediational patterns indicates that math self-efficacy and academic utility value—as measured here—may relate to STEM degree attainment through different pathways. Utility value was associated with STEM outcomes almost entirely through the shared variance it holds with educational expectations, whereas math self-efficacy retained a substantial association with STEM attainment that was independent of those expectations. This pattern is consistent with the hierarchical organisation of motivational beliefs posited by situated expectancy-value theory (Eccles and Wigfield, 2020), in which broader motivational orientations are linked to specific choices through a chain of increasingly proximal beliefs and expectations. It also complements prior work documenting the importance of domain-specific competence beliefs for STEM participation (Toh and Watt, 2022; Jiang et al., 2020; Gaspard et al., 2019; Fong et al., 2021) and research showing that the predictive power of generalised motivational beliefs for domain-specific outcomes can depend on more proximal cognitive mediators (Guo et al., 2015). Because the utility value items available in ELS:2002 refer to education in general, the full mediation pattern observed here may partly reflect the generalised nature of the measure rather than a property of utility value per se; this possibility cannot be ruled out with the present data.

One speculative interpretation of this pattern, consistent with the multinomial logit results reported in the Supplementary material, is that generalised academic utility value is most relevant to the initial decision to pursue higher education, rather than to the choice of a specific field of study once enrolled. In the multinomial analysis, utility value significantly distinguished students who never attended college from those who earned a non-STEM degree, but did not distinguish STEM from non-STEM degree earners among college entrants. Domain-specific math self-efficacy, by contrast, distinguished STEM from non-STEM completers, suggesting a more focal role in discipline-specific selection. This interpretation aligns with the hierarchical structure of motivated decision-making posited by situated expectancy-value theory (Eccles and Wigfield, 2020). Whether this distinction reflects a genuine functional property of generalised versus domain-specific motivational beliefs, or whether it is an artefact of the domain anchoring of the utility value measure, remains an open question for research using matched measures at both levels of specificity. If this interpretation is correct, it would imply that the effects of utility-value interventions on STEM outcomes (e.g., Rozek et al., 2017) may partly depend on whether such interventions also influence students’ broader educational expectations—a possibility that has not been directly tested.

Beyond their implications for the present study, the measurement characteristics of the available items carry broader relevance for EVT research. The divergent mediational patterns reported here are consistent with the possibility that motivational beliefs anchored at different levels of specificity—general education versus a particular academic domain—may relate to educational outcomes through different pathways. If this is the case, then studies comparing expectancy and value constructs would benefit from measuring both at matched levels of domain specificity, as is already standard practice in many EVT studies (cf. Bandura, 2006). Without such alignment, divergent patterns may reflect differences in the targeting of the items in addition to, or rather than, genuine functional differences between the constructs themselves. Future applications of the present longitudinal mediation framework with domain-matched measures of both expectancy and value would be positioned to determine whether the patterns observed here are replicable when measurement specificity is held constant.

### Educational expectations and family background

Family SES was the strongest predictor of educational expectations (*b* = 0.701), yet showed no significant direct association with STEM degree attainment after accounting for educational expectations and motivational beliefs (*b* = −0.004, *p* = 0.944). This pattern is consistent with the view that educational expectations represent one pathway through which family background is associated with educational outcomes (Ripamonti, 2025; Rangel et al., 2020), though it does not imply that SES is associated with STEM outcomes only through expectations—family resources may also operate through unmeasured channels such as social networks and cultural capital. The finding suggests that interventions designed to raise educational expectations may help reduce SES-based disparities in STEM participation, consistent with experimental evidence on the lasting benefits of brief psychological interventions targeting academic beliefs (Yeager et al., 2019; Walton and Cohen, 2011). The present findings extend prior evidence that educational expectations mediate the link between family background and academic attainment (Yong et al., 2026; Hofer et al., 2024) by demonstrating that expectations similarly channel the effects of motivational beliefs on STEM degree attainment. Whether interventions designed to raise educational expectations can reduce socioeconomic disparities in STEM participation is a hypothesis that warrants direct experimental evaluation.

### Gender differences

Research on gender disparities in STEM has documented that women’s underrepresentation is shaped by a confluence of factors, including lower math self-efficacy, different career interests, perceived work–family conflict, and implicit bias in educational and workplace contexts (Wang and Degol, 2017; Cheryan et al., 2017). Within this complex landscape, the present finding that educational expectations are a particularly strong predictor of STEM degree attainment for female students (*b* = 0.668 for females vs. *b* = 0.248 for males, interaction *p* < 0.001) is noteworthy. It suggests that, for women, the cognitive representation of one’s educational future may carry particular weight in determining whether they enter and complete a STEM degree—perhaps because high educational expectations serve as a countervailing force against the multiple barriers identified in prior work. This interpretation is consistent with Dangur-Levy's (2026) finding that math self-efficacy partially mediates the gender gap in STEM education, and with Jiang and Simpkins’ (2024) evidence that motivational beliefs interact with gender and college generation status in predicting STEM choices. While the present observational data cannot establish causality, this pattern raises the possibility that educational expectations may serve as a particularly important protective factor for female students’ STEM participation—a hypothesis that requires direct experimental investigation.

### Limitations and future directions

The utility value measure captured only the instrumental dimension of task value and was anchored to education generally rather than to mathematics specifically—a measurement limitation discussed throughout the paper. The STEM degree outcome was defined using the SMART Grant classification; alternative schemes could yield different results. The sample was restricted to U.S. students who were in Grade 10 in 2002; generalisability to other cohorts or educational systems is uncertain. Although FIML provided consistent estimates under the MAR assumption, the strong association between baseline achievement and STEM degree missingness (*d* = 0.80) raises the possibility of non-random missingness, and statistical adjustment cannot definitively rule out this concern. Finally, the binary STEM degree outcome collapses distinctions among STEM subfields; future work could examine whether the patterns identified here differ across engineering, life sciences, physical sciences, and computer science.

## Conclusion

This study examined how adolescent math self-efficacy and academic utility value are associated with STEM degree attainment a decade later, using a nationally representative U.S. longitudinal sample. The findings indicate that generalised academic utility value is associated with STEM outcomes almost entirely through educational expectations, whereas domain-specific math self-efficacy retains a substantial direct association with STEM attainment. These divergent patterns, interpreted with due recognition of the confounding between domain anchoring and the expectancy–value distinction, are consistent with the view that motivational beliefs anchored at different levels of specificity may relate to STEM outcomes through different pathways. However, the extent to which this reflects genuine psychological differences between expectancy and value constructs, as opposed to differences in the domain specificity of the available measures, remains an open question for future research with matched measurement instruments. Educational expectations also served as a significant pathway linking family SES to STEM outcomes and were a particularly strong predictor of STEM attainment for female students. A supplementary analysis including Grade 10 educational expectations as a covariate left all core findings unchanged, providing additional support for the temporal ordering of the mediation chain. These findings suggest that efforts to broaden STEM participation may benefit from addressing both students’ domain-specific competence beliefs and their broader educational ambitions.

## Data Availability

Publicly available datasets were analyzed in this study. This data can be found here: https://nces.ed.gov/surveys/els2002/.
