# Supplementary material for: Domain-specific and generalised motivational pathways to STEM degree attainment: a 10-year longitudinal mediation analysis
Source: Front Psychol. 2026 Aug 17;17:1898011. doi: 10.3389/fpsyg.2026.1898011 (PMC13526589; doi:10.3389/fpsyg.2026.1898011)
Supplement: Supplementary file 1 [file Supplementary_file_1.pdf]

Supplementary Material

Domain-Specific and Generalised Motivational Pathways to STEM Degree Attainment: A 10-Year Longitudinal Mediation Analysis

Table S1. Nested Model Comparisons — Core Path Coefficients Across Covariate Specifications

Path	M1: Core	M2: + Ability/SES	M3: Full
EdExp (ordered logit)			
MathSE → EdExp	.360*** (11.81)	.025 (0.79)	.114*** (3.55)
UtilVal → EdExp	.580*** (17.35)	.621*** (18.40)	.539*** (16.04)
z_math → EdExp	—	.670*** (35.60)	.658*** (31.63)
z_ses → EdExp	—	.246*** (14.62)	.701*** (25.42)
STEM degree (logit)			
EdExp → STEM degree	.461*** (13.38)	.355*** (9.71)	.389*** (10.00)
MathSE → STEM degree (direct)	.807*** (12.82)	.441*** (6.84)	.370*** (5.61)
UtilVal → STEM degree (direct)	−.111 (−1.70)	.009 (0.13)	.037 (0.55)
z_math → STEM degree	—	.763*** (16.68)	.704*** (14.07)
z_ses → STEM degree	—	−.087** (−2.60)	−.004 (−0.07)
Model information			
Gender and race/ethnicity included	No	No	Yes
Latent correlation (MathSE–UtilVal)	.46	.46	.46
N	15,355	15,117	14,527

Note. Logit coefficients from generalised structural equation models with ordered-logit (EdExp) and logit (STEM degree) links. z-statistics in parentheses. M1 = no covariates; M2 adds baseline math achievement (z_math) and family SES (z_ses); M3 (full) further adds gender and race/ethnicity. All models include residual covariances MSE1–MSE2 and MSE4–MSE5. The MathSE → EdExp path was not significant in M2 ($p = .428$), indicating sensitivity to covariate specification; this is discussed in the main text. The UtilVal → STEM degree path was

non-significant in all three models, consistent with full mediation. *p < .05, **p < .01, ***p < .001.

Table S2. Weighted (BRR) vs. Unweighted (GSEM) Path Coefficients

Path	Unweighted GSEM	Weighted BRR
EdExp		
MathSE → EdExp	.114*** (3.55)	.120*** (4.04)
UtilVal → EdExp	.539*** (16.04)	.362*** (14.22)
z_math → EdExp	.658*** (31.63)	.655*** (17.13)
z_ses → EdExp	.701*** (25.42)	.693*** (16.45)
Female → EdExp	.671*** (20.07)	.621*** (9.84)
STEM degree		
EdExp → STEM degree	.389*** (10.00)	.364*** (5.75)
MathSE → STEM degree (direct)	.370*** (5.61)	.273*** (4.32)
UtilVal → STEM degree (direct)	.037 (0.55)	.014 (0.27)
z_math → STEM degree	.704*** (14.07)	.674*** (9.16)
z_ses → STEM degree	−.004 (−0.07)	.030 (0.33)
Female → STEM degree	−.671*** (−9.13)	−.788*** (−5.38)
N	14,527	10,039

Note. Unweighted GSEM uses latent MathSE and UtilVal with FIML. Weighted model uses factor scores (means of respective items) with BRR estimation, F3BYPNLWT panel weights, and 200 BRR replicate weights (BYSTU1–BYSTU200). Both models include the same covariates. Weighted models exclude observations with weight = 0. The pattern of results is consistent across the two estimation methods, supporting the robustness of the findings to the complex survey design (Solon et al., 2015). t-statistics in parentheses for the weighted model (due to BRR variance estimation); z-statistics for the unweighted model. *p < .05, **p < .01, ***p < .001.

Table S3. Gender-Stratified Path Coefficients From Multigroup GSEM

Path	Male (n = 7,163)	Female (n = 7,364)
EdExp		
MathSE → EdExp	.119* (2.40)	.087* (2.01)
UtilVal → EdExp	.557*** (11.13)	.529*** (11.43)
z_math → EdExp	.656*** (22.73)	.660*** (21.90)
z_ses → EdExp	.814*** (20.24)	.599*** (15.82)
STEM degree		
EdExp → STEM degree	.248*** (5.24)	.668*** (9.56)
MathSE → STEM degree (direct)	.328*** (3.64)	.435*** (4.35)
UtilVal → STEM degree (direct)	.064 (0.74)	.007 (0.06)
z_math → STEM degree	.762*** (11.91)	.617*** (7.58)
z_ses → STEM degree	−.078 (−1.01)	.135 (1.46)
Model intercept (STEM equation)	−3.902	−7.611

Note. Logit coefficients from gender-stratified GSEMs. Both models include covariates (z_math, z_ses, race/ethnicity) and residual covariances (MSE1–MSE2, MSE4–MSE5). The non-significant direct effect of UtilVal on STEM degree in both groups replicates the full mediation pattern across gender. The Wald test comparing independent subgroup coefficients for the EdExp → STEM pathway yielded $z = 4.98$, $p < .001$. The formal interaction test is reported in Table S11.
z-statistics in parentheses. *p < .05, **p < .01, ***p < .001.

Table S4. Path Coefficients for the College-Attending Subsample

Path	Full sample	College-attending only
STEM degree (logit)		
EdExp → STEM degree	.389*** (10.00)	.328*** (7.76)
MathSE → STEM degree (direct)	.370*** (5.61)	.452*** (6.55)
UtilVal → STEM degree (direct)	.037 (0.55)	−.055 (−0.78)
z_math → STEM degree	.704*** (14.07)	.576*** (10.92)
z_ses → STEM degree	−.004 (−0.07)	−.171** (−2.81)
N	14,527	10,428

Note. Logit coefficients from GSEMs with identical specifications. The college-attending subsample excludes students who never attended any postsecondary institution (F3TZSTEM1CRED = −3). Both models include the full set of covariates and the measurement model for MathSE and UtilVal. The non-significant direct association of UtilVal with STEM degree in the college-attending subsample indicates that the full mediation pattern is not an artefact of college entry thresholds. The coefficient for z_ses became significantly negative in this subsample, a finding discussed in the main text. z-statistics in parentheses. *p < .05, **p < .01, ***p < .001.

Table S5. "Don't Know" Sensitivity — Alternative Treatments of EdExp

Path	FIML (primary)	Complete case	DK = 0
STEM degree (logit)			
EdExp → STEM degree	.389*** (10.00)	.389*** (10.01)	.208*** (7.30)
MathSE → STEM degree (direct)	.370*** (5.62)	.368*** (5.62)	.395*** (6.10)
UtilVal → STEM degree (direct)	.037 (0.59)	.040 (0.59)	.092 (1.40)
N	14,527	12,784	14,910

Note. FIML = full-information maximum likelihood (primary analysis). Complete case = observations with missing EdExp excluded. DK = 0 = "don't know" responses recoded as the lowest category (0) on a 0–8 scale. In all three specifications, the direct association of utility value with STEM degree remains non-significant. z-statistics in parentheses. *p < .05, **p < .01, ***p < .001.

Table S6. Predictors of Missingness — Logistic Regression Models (Odds Ratios)

Predictor	STEM degree missing	EdExp missing	MathSE missing
z_math	0.479*** (−31.30)	0.600*** (−19.68)	0.806*** (−10.58)
z_ses	0.515*** (−20.23)	0.784*** (−6.57)	0.835*** (−6.22)
Female (vs. male)	0.506*** (−17.12)	0.818*** (−4.42)	0.710*** (−9.59)
Black	0.883 (−1.62)	1.131 (1.44)	1.307*** (3.74)
Hispanic	0.999 (−0.02)	1.174* (2.43)	1.316*** (5.11)
Asian	0.725*** (−5.48)	0.910 (−1.40)	1.815*** (11.31)
Constant	0.545*** (−19.72)	0.192*** (−44.78)	0.476*** (−25.78)
N	15,244	15,244	15,244

Note. Odds ratios from logistic regression models predicting the probability that a given variable is missing (1 = missing, 0 = observed). OR > 1 indicates higher odds of missingness; OR < 1 indicates lower odds. z-statistics in parentheses. All models include the full set of covariates. Students with lower math achievement, lower SES, and male gender were significantly more likely to have missing data. Because these variables are included in all analytic models, the missing-at-random (MAR) assumption underlying FIML estimation is considered tenable (Enders, 2010; Graham, 2009). *p < .05, **p < .01, ***p < .001.

Table S7. E-Value Sensitivity Analysis Summary

Variable	b (logit)	OR	95% CI	E-value
EdExp → STEM	.386	1.472	[1.351, 1.602]	2.30
Math self-efficacy → STEM	.298	1.347	[1.230, 1.476]	2.03
Utility value → STEM	.037	1.038	[0.955, 1.128]	1.24

Note. E-values calculated from factor-score logistic regression models following VanderWeele and Ding (2017). The E-value represents the minimum strength of association, on the risk ratio scale, that an unmeasured confounder would need to have with both the exposure and the outcome to explain away the observed exposure–outcome association, conditional on the measured covariates. The factor-score logit model included z_math, z_ses, gender, and race/ethnicity as covariates. Because the direct association of utility value with STEM degree is not statistically different from zero, its E-value is reported for completeness but is not interpreted as evidence of robustness.

Table S8. Standard vs. Firth's Penalized Logistic Regression Coefficients

Variable	Standard logit	Firth's logit
EdExp	.386*** (8.89)	.385*** (8.87)
Math self-efficacy (z)	.298*** (6.41)	.297*** (6.41)
Utility value (z)	.037 (0.87)	.037 (0.87)
z_math	.651*** (11.59)	.649*** (11.57)
z_ses	−.013 (−0.20)	−.014 (−0.21)
Female	−.649*** (−7.95)	−.647*** (−7.94)
Black	−.268 (−1.16)	−.250 (−1.09)
Hispanic	−.299* (−1.98)	−.292 (−1.94)
Asian	.148 (0.95)	.154 (0.99)
Constant	−4.866*** (−16.93)	−4.854*** (−16.91)
N	7,581	7,581

Note. Firth's penalized maximum likelihood estimation provides bias correction for logistic regression with rare events (Peduzzi et al., 1996; Vittinghoff & McCulloch, 2007). Coefficients are logit coefficients; z-statistics in parentheses. The near-identical coefficients across standard and penalized estimation indicate that the approximately 7% event rate of STEM degree attainment did not introduce appreciable bias in the standard maximum likelihood estimates. *p < .05, **p < .01, ***p < .001.

Table S9. Multinomial Logistic Regression — Three-Category Outcome

Variable	No college vs. Non-STEM	STEM vs. Non-STEM
EdExp	−.194*** (−8.39)	.314*** (7.03)
Math self-efficacy (z)	.096** (3.05)	.329*** (6.93)
Utility value (z)	−.147*** (−4.93)	−.008 (−0.18)
z_math	−.411*** (−11.55)	.516*** (8.95)
z_ses	−.449*** (−10.04)	−.160* (−2.35)
Female (vs. male)	−.621*** (−11.25)	−.839*** (−10.06)
Black	.480*** (3.94)	−.048 (−0.20)
Hispanic	.340*** (3.98)	−.182 (−1.17)
Asian	.421*** (4.65)	.333* (2.07)
Constant	1.256*** (8.75)	−3.722*** (−12.57)
N	7,581	

Note. Coefficients are logit coefficients from a multinomial logistic regression model; z-statistics in parentheses. Reference outcome category is Non-STEM college degree. The first column contrasts students who never attended college with those who earned a non-STEM degree. The second column contrasts STEM degree earners with non-STEM degree earners. Utility value significantly predicts the college entry threshold (No college vs. Non-STEM) but does not significantly predict STEM-specific choice among college entrants (STEM vs. Non-STEM), consistent with the full mediation pattern reported in the main text. *p < .05, **p < .01, ***p < .001.

Table S10. KHB Decomposition of Direct, Indirect, and Rescaling Effects

Parameter	UtilVal → STEM	MathSE → STEM
Reduced model (total association)	.118** (2.82)	.325*** (7.01)
Full model (direct association)	.037 (0.87)	.298*** (6.41)
Difference (indirect association)	.081*** (7.64)	.027*** (4.13)
Confounding ratio	3.197	1.091
Rescaling factor	1.152	0.992

Note. KHB decomposition (Karlson, Holm, & Breen, 2012) applied to factor-score logit models with EdExp as the mediator and all covariates included as concomitants. The reduced model excludes the mediator; the full model includes it. The confounding ratio is the ratio of the reduced-model coefficient to the full-model coefficient; it quantifies the total attenuation of the coefficient attributable to both mediation and rescaling. The rescaling factor is the ratio of the full-model to reduced-model residual standard deviation; it isolates the portion of coefficient change due to the fixed residual variance of the logit model ($\pi^2/3$). The indirect association, net of rescaling, is given by the difference. For utility value, the rescaling factor was 1.15 and the indirect association remained significant, confirming that genuine mediation is present. For math self-efficacy, rescaling was negligible (0.99). z-statistics in parentheses. *$p < .05$, **$p < .01$, ***$p < .001$.

Table S11. Gender Interaction Test — Female × EdExp

Variable	b (SE)	z	p
EdExp	.236 (.051)	4.57	< .001
Female (vs. male)	−3.636 (.627)	−5.80	< .001
Female × EdExp	.428 (.088)	4.84	< .001
Math self-efficacy (z)	.298 (.046)	6.41	< .001
Utility value (z)	.038 (.042)	0.89	.371
z_math	.656 (.056)	11.66	< .001
z_ses	−.005 (.066)	−0.07	.943
Black	−.287 (.232)	−1.24	.215
Hispanic	−.295 (.151)	−1.95	.051
Asian	.146 (.156)	0.94	.348
Constant	−3.857 (.337)	−11.45	< .001
N	7,581		

Note. Logistic regression predicting STEM degree attainment in the full sample. The significant interaction term indicates that the EdExp → STEM pathway is stronger for females ($b = .236 + .428 = .664$) than for males ($b = .236$). The Wald test comparing independent subgroup coefficients from the gender-stratified GSEMs (Table S3) confirmed this finding: $z = 4.98$, $p < .001$. *$p < .05$, **$p < .01$, ***$p < .001$.
